# Development of CT Effective Dose Conversion Factors from Clinical CT Examinations in the Republic of Korea

**DOI:** 10.3390/diagnostics10090727

**Published:** 2020-09-21

**Authors:** Sang-Kyung Lee, Jung Su Kim, Sang-Wook Yoon, Jung Min Kim

**Affiliations:** 1 Health Science Research Center, Korea University, Seoul 02841, Korea; sk_lee@korea.ac.kr; 2 Department of Radiologic Technology, Daegu Health College, Daegu 41453, Korea; jungsu.kim73@gmail.com; 3 Department of Diagnostic Radiology, CHA Bundang Medical Center, CHA University, Pocheon-si 13496, Gyeonggi-do, Korea; 4 School of Health and Environmental Science, Korea University, Seoul 02841, Korea

**Keywords:** computed tomography, effective dose, dose-length product, dosimetry

## Abstract

The aim of this study was to determine the conversion factors for the effective dose (ED) per dose length product (DLP) for various computed tomography (CT) protocols based on the 2007 recommendations of the International Commission on Radiological Protection (ICRP). CT dose data from 369 CT scanners and 13,625 patients were collected through a nationwide survey. Data from 3793 patients with a difference in height within 5% of computational human phantoms were selected to calculate ED and DLP. The anatomical CT scan ranges for 11 scan protocols (adult-10, pediatric-1) were determined by experts, and scan lengths were obtained by matching scan ranges to computational phantoms. ED and DLP were calculated using the NCICT program. For each CT protocol, ED/DLP conversion factors were calculated from ED and DLP. Estimated ED conversion factors were 0.00172, 0.00751, 0.00858, 0.01843, 0.01103, 0.02532, 0.01794, 0.02811, 0.02815, 0.02175, 0.00626, 0.00458, 0.00308, and 0.00233 mSv∙mGy^−1^∙cm^−1^ for the adult brain, intra-cranial angiography, C-spine, L-spine, neck, chest, abdomen and pelvis, coronary angiography, calcium scoring, aortography, and CT examinations of pediatric brain of <2 years, 4–6 years, 9–11 years, and 13–15 years, respectively. We determined ED conversion factors for 11 CT protocols using CT data obtained from a nationwide survey in Korea and Monte Carlo-based dose calculations.

## 1. Introduction

Advances in computed tomography (CT) have led to an increase in its diagnostic capabilities and therefore its utilization, which in turn has led to an increase in patient exposure to radiation. Diagnostic reference levels apply to the radiation exposure of patients resulting from procedures performed for medical imaging and are used in medical imaging to determine whether the radiation dose was unusually high or low for a specific procedure. Alternatively, effective dose (ED) can be beneficial to compare the relative doses of different diagnostic procedures and technologies in different hospitals and countries. Furthermore, ED can also be used to study the use of different technologies for the same medical examination. However, it should be noted that ED is not used to determine individual risk [1]. Thus, calculations of ED received by the patient during CT examinations can provide meaningful information.

According to the International Commission on Radiological Protection (ICRP), ED represents a weighted sum of the equivalent doses in all tissues and organs of the body, where the equivalent doses for an organ represent the sum of absorbed doses averaged over a tissue or an organ weighted by the radiation weighting factor. Generally, ED is computed by using Monte Carlo dose simulation tools for reference human phantoms. The ED evaluation method for CT examinations in the clinical setting has been defined using dose-length product (DLP) and the effective dose conversion factor (ED/DLP). In the year 1999, the European Commission published a set of conversion factors for different regions of the adult body [2]. Shrimpton [3] extended the conversion factor computation to pediatric patients. In the ICRP publication 102 [4], the k-factor was introduced. The k-factors are only used for six scan areas (head and neck, head, neck, chest, abdomen and pelvis, and trunk). The published conversion factors did not reflect differences between hospitals and individuals in various countries and were computed with outdated hermaphrodite-stylized human phantoms based on the previous ICRP recommendations [5] regarding ED. Therefore, ED conversion factors based on the new ICRP recommendations in publication 103 are needed for various CT protocols for adult and pediatric patients. 

In this study, we developed CT ED conversion factors for 11 CT protocols, utilizing a nationwide survey data conducted in the Republic of Korea. We used the Monte Carlo-based dose calculation program, which adopts hybrid computational phantoms representing individuals with a reference organ mass and body dimensions ranging from newborns to adults, including six age groups that include children and both sexes [6].

## 2. Materials and Methods 

### 2.1. CT Examination Data Acquisition

Data required for the calculation of ED conversion factors were extracted from data collected through a nationwide survey carried out from October 2016 to August 2017 in the Republic of Korea [7]. The survey protocol was selected after conducting a large data analysis of the 2015 National Health Insurance Service Data. The adult CT protocol was the most frequent CT examination. Moreover, the pediatric brain CT protocol was included as pediatric patients are more sensitive to radiation exposure than adult patients. We selected 11 CT protocols for our study, including brain CT, intra-cranial CT angiography, cervical spine CT, lumbar spine CT, neck CT, chest CT, abdomen-pelvis CT, coronary artery CT angiography, coronary artery CT calcium score, CT aortography, and pediatric brain CT. The survey complied with the Health Insurance and Portability and Accountability Act and was prospectively approved by the institutional review boards (CHAMC 2016-12-006, 5 December 2016), which waived the need for written informed consent.

We assumed that the physical characteristics of the patients would be similar to those of the virtual phantoms used for the dosimetry calculator and established the following conditions for extracting patient data from the survey: (1) data of adult patients with heights in the following ranges: 169–187 cm for men and 159–176 cm for women; (2) data of pediatric patients belonging to the four age groups as follows: 0–2, 4–6, 9–11, and 13–15 years. We extracted data for a total of 3793 CT examinations for this study, which included examinations of 1942 adult men, 1107 adult women, and 744 pediatric patients. The number of examinations for each CT protocol is listed in Table 1. The characteristics of the adult patients are summarized in Table 2 and Table 3.

### 2.2. Dosimetry Calculator

The CT ED conversion factor is defined as ED per DLP. Therefore, DLP and ED must be calculated for each CT examination. Since ED is not a physical quantity that can be measured, a CT dosimetry calculation program is required. In this study, we used the NCICT (National Cancer Institute dosimetry system for Computed Tomography) program (Available online: http://ncidose.cancer.gov/#ncict; accessed on 10 September 2020) based on a Monte Carlo simulation, which was developed by the U.S. National Cancer Institute [8]. The NCICT program uses computational human phantoms in the latest dosimetry calculator for evaluating ED based on the 2007 recommendations of the ICRP. The radiation weighting factor is 1 for photons and electrons. The tissue weighting factor values for organs and tissues are shown in Table 4.

### 2.3. Calculations of DLP and ED

**Figure 1:**
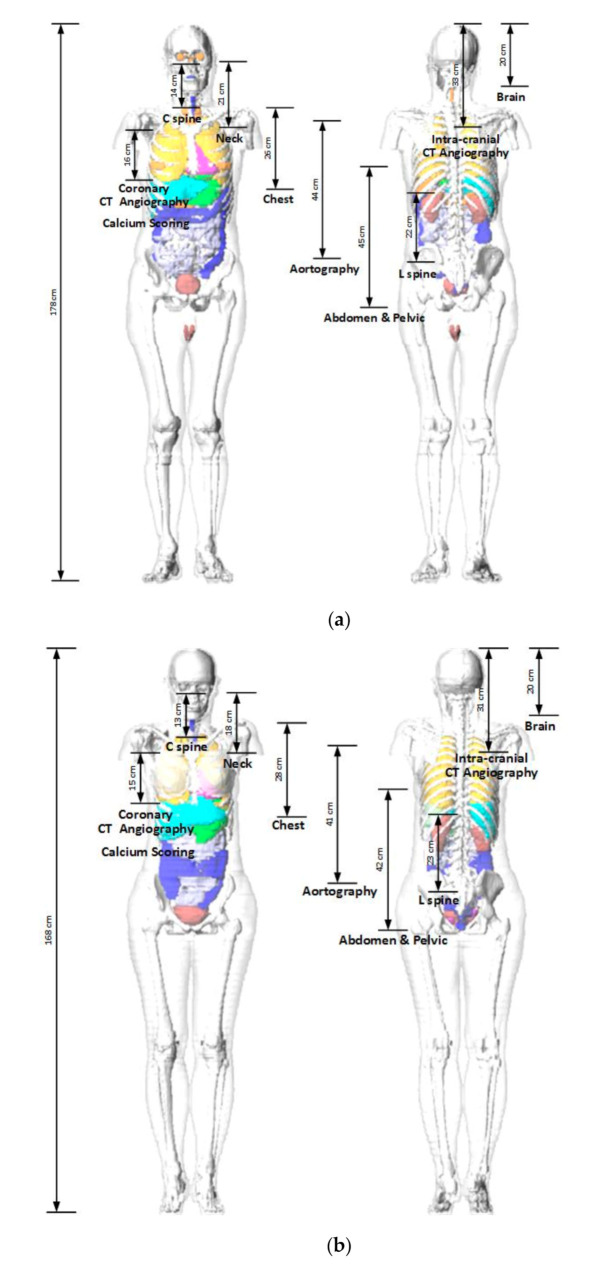
Adult phantoms used in the NCICT dose calculation program. The measurements show the relationship between the scanning ranges of 10 computed tomography examinations and organ positions (Figures of the phantoms were extracted from the NCICT program): (**a**) male; (**b**) female.

**Figure 2:**
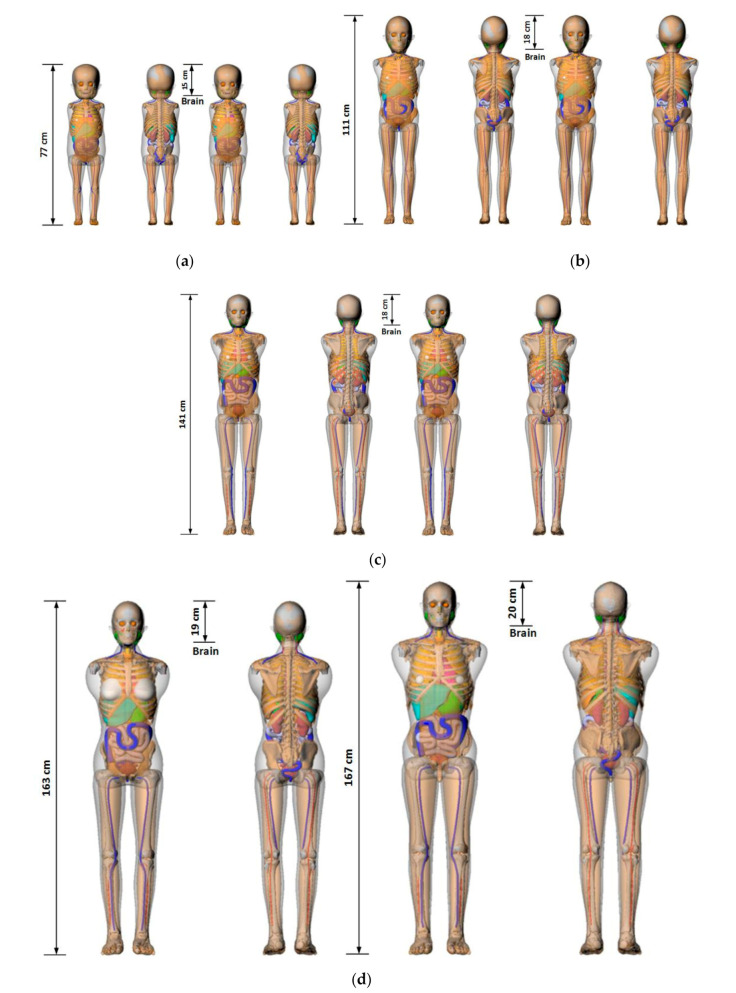
Pediatric phantoms used in the NCICT dose calculation program. The measurements show the relationship between the scanning ranges of 10 computed tomography examinations and organ positions (Figures of the phantoms were extracted from the NCICT program). Images of (**a**) 1-year-old female (left) and male (right) phantom, (**b**) 5-year-old female (left) and male (right) phantom, (**c**) 10-year-old female (left) and male (right) phantom, and (**d**) 15-year-old female (left) and male (right) phantoms are shown.

To calculate the ED received by a patient from a CT examination using the NCICT program, information containing CTDI_vol_, age, tube voltage, scan range, sex, and type of head/body filter is needed. The scan length required for dose calculations in the NCICT program was set through the anatomical scan range for each CT protocol determined by radiologists (Table 5). The anatomical scan range was applied to computational human phantoms used in the NCICT program for newborns, individuals aged 0, 1, 5, 10, and 15 years, and adult male and female reference individuals whose organ mass matches the values listed in Publication 89 of the ICRP [9]. We set scan ranges required for the dose calculation. For 10 adult CT protocols, the scan ranges were marked in the computational human phantom picture of a male and a female adult (Figure 1). For pediatric patients in the four age groups, computational human phantoms for ages 1, 5, 10, and 15 years were used, respectively, and the scan lengths for each brain protocol are shown in Figure 2. Based on the scan length set, DLP and ED can be calculated for each patient through the NCICT program. Previously, Christner [10] set the scan length to 13.5, 27.5, 12.5, 26.5, and 45.5 cm for five CT scan protocols (head, chest, coronary, liver, and abdomen-pelvis, respectively) when performing dose calculations using the ImPACT calculator (Company name, Available online: website; accessed on Day Month Year) [11].

## 3. Results

### 3.1. Statistical Analysis of CT Examination Data

**Figure 3:**
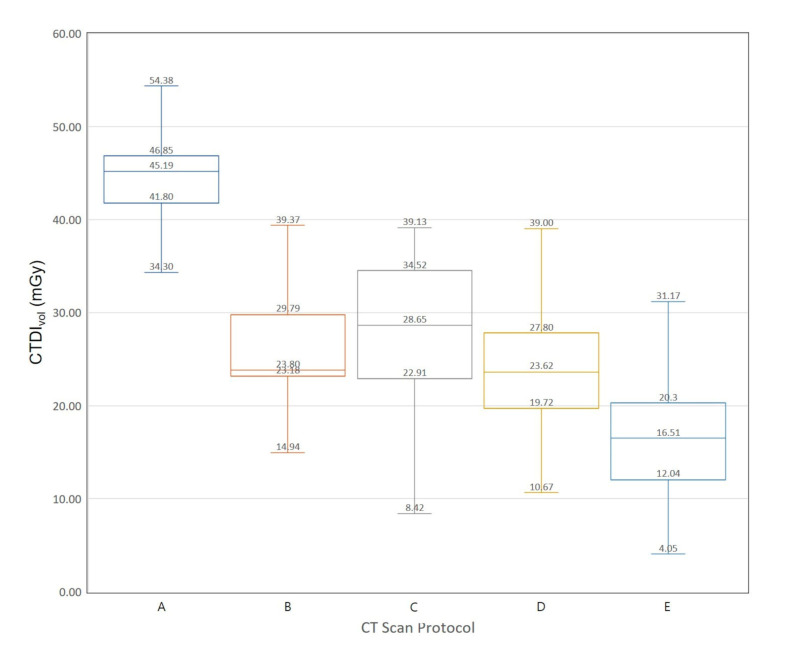
Box and whisker plot of CTDI_vol_ for brain computed tomography examination of male patients. **A:** Adult, **B:** pediatric patients, 13–15 years of age, **C:** pediatric patients, 9–11 years of age, **D:** pediatric patients, 4–6 years of age, **E:** pediatric patients, <2 years of age.

**Figure 4:**
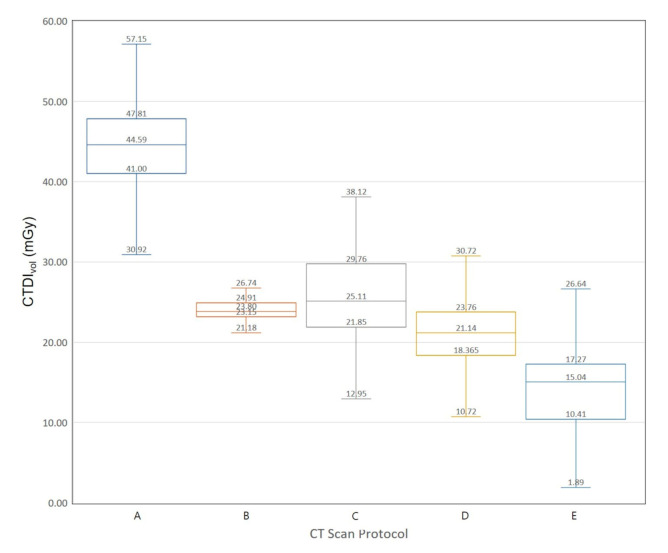
Box and whisker plot of CTDI_vol_ for brain computed tomography examination of female patients. **A:** Adult, **B:** pediatric patients, 13–15 years of age, **C:** pediatric patients, 9–11 years of age, **D:** pediatric patients, 4–6 years of age, **E:** pediatric patients, <2 years of age.

**Figure 5:**
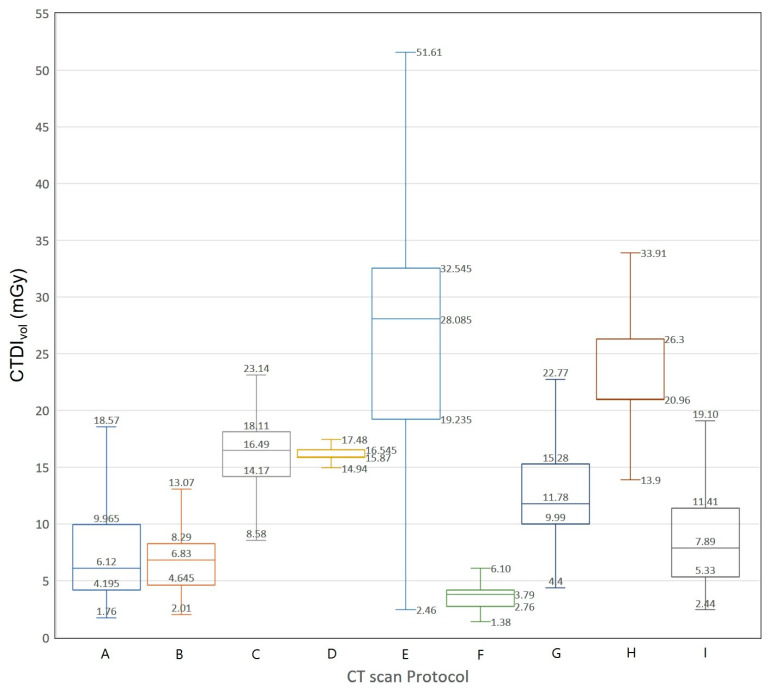
Box and whisker plot of CTDI_vol_ for computed tomography examinations (except for brain CT examination) of adult male patients. **A:** abdomen and pelvis, **B:** chest, **C:** cervical spine, **D:** lumbar spine, **E:** coronary angiography, **F:** calcium scoring, **G:** neck, **H:** intra-cranial angiography, **I:** aortography.

**Figure 6:**
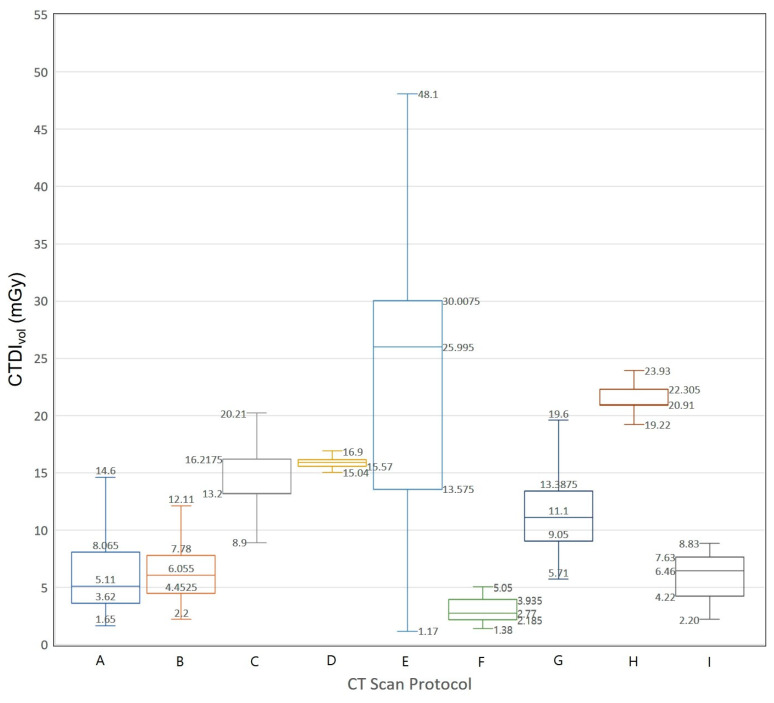
Box and whisker plot of CTDI_vol_ for computed tomography examinations (except for brain CT examination) of adult female patients. **A:** abdomen and pelvis, **B:** chest, **C:** cervical spine, **D:** lumbar spine, **E:** coronary angiography, **F:** calcium scoring, **G:** neck, **H:** intra-cranial angiography, **I:** aortography.

We extracted data on 3793 CT examinations from a nationwide survey data in Korea. Figure 3 and Figure 4 show Box-Whisker plots of CTDI_vol_ for brain CT examinations of adult and pediatric patients. The 75th percentile values of CTDI_vol_ for male brain CT examinations are 46.85, 29.79, 34.52, 27.80, and 20.30 mGy. The 75th percentile values of CTDI_vol_ for female brain CT examinations are 47.81, 24.91, 29.76, 23.76, and 17.27 mGy, respectively. Figure 5 and Figure 6 show Box-Whisker plots of CTDI_vol_ for adult CT examinations of the chest, abdomen, and other body parts. The 75th percentile values of CTDI_vol_ for male and female patients, respectively, were as follows: abdomen-pelvic, 9.97 and 8.07 mGy; chest, 8.29 and 7.78 mGy; cervical spine, 18.11 and 16.22 mGy; lumbar spine, 16.55 and 16.13 mGy; coronary angiography, 32.55 and 30.01 mGy; calcium scoring, 4.20 and 3.94 mGy; neck, 15.28 and 13.39 mGy; intra-cranial angiography, 26.30 and 22.31 mGy; and aortography, 11.41 and 7.63 mGy. Table 6 shows the average tube voltages for each CT examination. The eleven CT protocols all used similar tube voltages in a range between 106 and 120 kV_p_.

The scan length values for adult CT examinations obtained from the survey are presented in Table 7. Among 10 CT examinations, the 75th percentile DLP value was the highest for the intra-cranial angiography CT examination and the lowest for the calcium scoring CT examination. Upon comparing 75th percentile values of scan length for adult CT examinations to scan lengths applied in the NCICT dose calculations, the relative errors of scan length for male and female CT examinations, respectively, were as follows: brain, 0.16 and 0.142; intra-cranial angiography, 0.218 and 0.229; cervical spine, 0.446 and 0.460; lumbar spine, 0.329 and 0.272; neck, 0.409 and 0.450; chest, 0.416 and 0.317; abdomen-pelvic, 0.245 and 0.238; coronary angiography, 0.085 and 0.092; calcium scoring, 0.059 and 0.094; and aortography, 0.470 and 0.459.

### 3.2. Estimation of CT ED Conversion Factors

For 3793 cases of CT examinations extracted from a nationwide survey data in Korea, DLP and ED were calculated for every patient using the NCICT program, and the average and standard deviation (SD) values for 11 CT protocols are summarized in Table 8 and Table 9.

The estimated ED conversion factors are summarized in Table 10, and the sex-averaged values of the ED conversion factor are as follows: 0.00172, 0.00751, 0.00858, 0.01843, 0.01103, 0.02532, 0.01794, 0.02811, 0.02815, 0.02175, 0.00626, 0.00458, 0.00308, and 0.00233 mSv∙mGy^−1^∙cm^−1^ for adult brain, intra-cranial angiography, C-spine, L-spine, neck, chest, abdomen & pelvis, coronary angiography, calcium scoring, aortography, and CT examinations of pediatric brain of <2 years, 4–6 years, 9–11 years, and 13–15 years, respectively.

Compared to previous studies [2,3,12,13] of the same CT protocols for adults, the ED conversion factor for the brain examination was 0.00172, which was lower than those previously reported: 0.0021 [3] and 0.0028 [12]. The ED conversion factor for the neck examination was 0.01103, which is 12.6% higher than the results from the 1999 German survey (0.0098) [12], and the ED conversion factor for the chest examination was 0.02532, which is higher than the results of previous studies: 0.014 [3], 0.0152 [12], and 0.017 [2]. In the abdomen-pelvis examination, the ED conversion factor was 0.01794, which is similar to the result of other studies: 0.015 [2] and 0.0174 [12]. The ED conversion factor for the pediatric brain examination was similar to that of previous studies. The ED conversion factors for the pediatric brain examination of children aged 1, 5, and 10 years reported in two previous studies [3,13] were 0.0067, 0.008, and 0.004, 0.003, and 0.003, respectively. These results were similar to the results of this study: 0.00626, 0.00458, and 0.00308.

## 4. Discussion

Recently most of the CT scanners report the DLP. Although the DLP is related to patient dose and risk, it is unique to CT and is not useful for comparisons with other modalities. DLP to ED conversion factors are useful for quickly estimating patient risk based on the DLP values reported by CT systems [14].

In the present work, we developed DLP to ED conversion factors for a greater number of CT protocols than those used in prior studies. These conversion factors are necessary for the current medical fields where various CT protocols are used. We calculated DLP and ED received by a patient from a CT examination using the Monte Carlo-based NCICT program with CT examination data on CTDI_vol_, age, tube voltage, scan range, sex, and type of head/body filter. The data required for the calculation of ED conversion factors were extracted from data collected in a nationwide survey in the Republic of Korea. The data of patients similar in size to the computational phantom size were used when calculating ED so that it was possible to estimate a proximal value of ED received by the patient.

In addition to the four CT protocols (brain, neck, chest, and abdomen-pelvis) used in the previous study, our study presented conversion factors for six additional CT protocols (cervical spine, lumbar spine, coronary angiography, calcium scoring, intra-cranial angiography, and aortography). In our study, ED conversion factors for neck and chest protocols are higher than those used in previous studies. This is related to the differences in organ-weighting factors and ED calculation between the ICRP publications 60 and 103. For the ICRP publication 60, the highest organ-weighting factors were found for the pelvic region, whereas for the ICRP publication 103, the highest values were obtained for the abdominal region. The conversion factors for the brain CT protocol for children are higher than those for adults. This result was expected, since the organs (lung, breast, and stomach) responsible for the main contribution to ED receive higher doses in children than the organs of adults due to their smaller cross sections [15]. The comparison of the conversion factors for males and females showed that higher conversion factors were obtained for adult females. We did not evaluate the conversion factor’s dependence on tube voltage because the variation by tube voltage can be neglected for all scanned regions [15].

CTDI_vol_ represents x-ray output from a CT scanner. However, the average conversion factor does not provide scanner-specific results. The ED is an estimate of relative biologic risk and is not a physical parameter that can be measured. Indeed, the ED is a derived parameter and is always computed through multiple steps and approximations. Martin [16] reported that the inherent relative uncertainties in estimating ED (using organ doses) received by a reference patient was about ±40%. The conversion factors provide clinicians and physicists with a quick and relatively accurate method to estimate ED and patient risk. However, the ED should be used with care. It does not represent the risk that exists for any particular patient. However, it is a useful concept for dose optimization, comparing technologies and modalities, and for population-based dose surveys and risk assessments [16]. Developing ED conversion factors of suitable scan protocols may improve the practicality of ED estimation, which is essential for the dose index registry system. Further research on the calculation of the ED conversion factor based on the patient’s body mass index could greatly assist in managing the exposure dose in the clinical setting.

## 5. Conclusions

We determine ED conversion factors that reflect the new ICRP recommendations. We specified these factors separately for both sexes for 11 CT examination protocols. These factors were based on CT examination data obtained from a nationwide survey in the Republic of Korea and developed using the Monte Carlo-based dose calculation program. These CT protocol-specific conversion factors will allow for a more accurate benefit-risk assessment of CT imaging strategies and optimization of patient radiation safety.

## Figures and Tables

**Table 1:** Number of patients to calculate the effective dose conversion factor.

CT Protocols	Number of Patients		
	Male	Female	Total
Brain	443	247	690
Intra-cranial angiography	119	108	227
C spine	187	74	261
L spine	177	85	262
Neck	111	70	181
Chest	217	126	343
Abdomen & pelvis	313	285	598
Coronary angiography	158	52	210
Calcium scoring	173	49	222
Aortography	44	11	55
Pediatric brain (<2 years)	175	163	338
Pediatric brain (4–6 years)	146	82	228
Pediatric brain (9–11 years)	94	35	129
Pediatric brain (13–15 years)	24	25	49
Total	2381	1412	3793

**Table 2:** The average age, height, and weight of adult male patients who underwent computed tomography.

CT Protocols	Age (Years)		Height (cm)		Weight (kg)	
	Average	SD	Average	SD	Average	SD
Brain	49	15.0	174.22	3.79	75.00	10.82
Intra-cranial angiography	50	14.2	174.00	3.69	73.08	11.51
C spine	44	12.1	175.00	4.02	75.30	8.92
L spine	45	13.6	175.00	4.28	74.96	8.38
Neck	53	13.9	174.00	4.05	72.19	9.17
Chest	55	16.3	173.53	3.67	71.08	9.41
Abdomen and pelvis	52	14.8	173.98	3.72	72.71	11.17
Coronary angiography	55	12.6	174.00	3.51	76.16	9.70
Calcium scoring	53	12.6	174.00	3.79	76.39	9.49
Aortography	59	14.5	173.00	3.38	74.92	9.59

**Table 3:** The average age, height, and weight of adult female patients who underwent computed tomography.

CT Protocols	Age (Years)		Height (cm)		Weight (kg)	
	Average	SD	Average	SD	Average	SD
Brain	46	13.6	163.00	3.33	59.13	9.55
Intra-cranial angiography	49	13.9	163.00	3.19	60.38	8.69
C spine	44	11.4	164.00	3.82	58.68	7.72
L spine	46	13.4	163.00	3.68	58.61	9.06
Neck	43	13.9	163.00	3.39	59.70	10.04
Chest	54	13.9	162.47	2.96	60.48	8.09
Abdomen and pelvis	47	14.3	162.90	3.28	59.46	9.93
Coronary angiography	57	10.4	162.00	2.64	62.69	9.63
Calcium scoring	57	11.7	163.00	2.89	62.02	8.99
Aortography	57	14.8	162.00	2.41	58.91	5.76

**Table 4:** Tissue weighting factors recommended by the International Commission on Radiological Protection (ICRP) in 1991 and 2007. The tissues in parentheses mean the remainder tissues. The tissue weighting factor for the remainder tissues applies to the arithmetic mean dose of the organs for each sex.

Tissue or Organ	ICRP 60	ICRP 103
Red bone marrow	0.12	0.12
Colon	0.12	0.12
Lung	0.12	0.12
Stomach	0.12	0.12
Breast	0.05	0.12
Gonads	0.20	0.08
Bladder	0.05	0.04
Esophagus	0.05	0.04
Liver	0.05	0.04
Thyroid	0.05	0.04
Bone surface	0.01	0.01
Brain	See below	0.01
Salivary glands	none	0.01
Skin	0.01	0.01
Remainder	0.05	0.12
(Adrenals)	(0.005)	(0.0086)
(Extra thoracic region)	none	(0.0086)
(Gall bladder)	none	(0.0086)
(Heart)	none	(0.0086)
(Kidneys)	(0.005)	(0.0086)
(Lymphatic nodes)	none	(0.0086)
(Muscle)	(0.005)	(0.0086)
(Oral mucosa)	none	(0.0086)
(Pancreas)	(0.005)	(0.0086)
(Small intestine)	(0.005)	(0.0086)
(Upper large intestine)	(0.005)	none
(Spleen)	(0.005)	(0.0086)
(Thymus)	(0.005)	(0.0086)
(Brain)	(0.005)	See above
(Prostate for male)	none	(0.0086)
(Uterus/cervix for female)	(0.005)	(0.0086)

**Table 5:** Anatomical scan ranges for the computed tomography (CT) protocols.

CT Protocols	Scan Start	Scan End
Brain	Skull base	Skull vertex
Intra-cranial angiography	1 cm below carotid artery branch	Skull vertex
C spine	1 cm above C spine No.1	C spine No.7
L spine	L spine No.1	Sacrum No.1
Neck	Sella turcica	Sternal notch
Chest	Lung apex	Adrenal glands
Abdomen and pelvis	Diaphragm	Pubic symphysis
Coronary angiography	1 cm above coronary artery branch	1 cm below heart apex
Calcium scoring		
Aortography	1 cm above aortic arch	Iliac artery bifurcation
Pediatric brain (<2 years)	Skull base	Skull vertex
Pediatric brain (4–6 years)		
Pediatric brain (9–11 years)		
Pediatric brain (13–15 years)		

**Table 6:** Tube voltage for computed tomography examinations of patients.

CT Protocols	Tube Voltage (kV_p_)	
	Average	SD
Brain	120	5.42
Intra-cranial angiography	106	9.88
C spine	120	4.89
L spine	120	3.83
Neck	118	6.35
Chest	119	5.89
Abdomen and pelvis	110	10.39
Coronary angiography	108	10.98
Calcium scoring	114	9.36
Aortography	114	9.28
Pediatric brain (<2 years)	111	11.82
Pediatric brain (4–6 years)	115	8.55
Pediatric brain (9–11 years)	117	9.10
Pediatric brain (13–15 years)	118	7.89

**Table 7:** Scan length (mm) for computed tomography examinations of adult patients.

CT Protocols	Male			Female		
	25th Percentile	Average	75th Percentile	25th Percentile	Average	75th Percentile
Brain	160.93	175.54	180.80	153.25	164.02	175.14
Intra-cranial angiography	180.00	324.29	421.94	199.73	326.83	401.92
C spine	214.92	233.67	252.66	200.00	228.19	240.78
L spine	291.09	311.95	328.03	282.97	306.19	316.09
Neck	310.00	329.87	355.62	274.75	321.68	327.34
Chest	379.20	416.45	445.26	350.00	377.87	410.07
Abdomen and pelvis	496.20	541.51	596.20	485.00	505.71	551.32
Coronary angiography	151.88	167.03	174.84	139.38	153.05	165.16
Calcium scoring	140.00	162.75	170.00	140.00	149.45	165.62
Aortography	690.00	741.95	829.75	571.60	649.54	757.66

**Table 8:** The dose-length product (mGy∙cm) calculated by the NCICT program.

CT Protocols	Male		Female	
	Average	SD	Average	SD
Brain	854.46	131.68	845.96	136.65
Intra-cranial angiography	908.91	1094.49	706.72	540.64
C spine	236.81	127.72	199.28	132.08
L spine	365.94	162.35	352.28	126.24
Neck	274.64	120.04	199.69	79.34
Chest	188.41	155.18	184.48	102.65
Abdomen and pelvis	331.62	283.77	259.95	198.50
Coronary angiography	445.05	365.88	327.83	183.20
Calcium scoring	56.48	26.63	45.33	21.26
Aortography	452.71	635.36	247.35	85.52
Pediatric brain (<2 years)	233.65	101.70	208.00	82.75
Pediatric brain (4–6 years)	410.79	129.41	369.44	101.22
Pediatric brain (9–11 years)	493.07	178.24	426.57	107.30
Pediatric brain (13–15 years)	488.89	130.44	449.22	172.16

**Table 9:** The effective dose (mSv) calculated by the NCICT program.

CT Protocols	Male		Female	
	Average	SD	Average	SD
Brain	1.3472	0.2118	1.6713	0.2761
Intra-cranial angiography	6.0912	7.4315	5.9761	4.6533
C spine	1.7257	0.9316	2.3582	1.5636
L spine	6.1910	2.7488	7.6019	2.7241
Neck	2.6284	1.1528	2.6652	1.0598
Chest	4.5806	3.7792	4.9947	2.7844
Abdomen and pelvis	5.1620	4.4824	5.3995	4.1718
Coronary angiography	11.908	9.9325	10.692	6.0320
Calcium scoring	1.5212	0.7267	1.4856	0.7010
Aortography	9.4329	12.9586	6.2414	2.2022
Pediatric brain (<2 years)	1.4604	0.6408	1.3098	0.5216
Pediatric brain (4–6 years)	1.8833	0.6004	1.6772	0.4536
Pediatric brain (9–11 years)	1.5276	0.5520	1.2936	0.3242
Pediatric brain (13–15 years)	1.1692	0.3082	1.0161	0.4004

**Table 10:** Conversion factors (mSv∙mGy^−1^∙cm^−1^) for dose-length product to effective dose.

CT Protocols	Male		Female	
	Average	SD	Average	SD
Brain	0.0016	0.000026	0.0020	0.000029
Intra-cranial angiography	0.0067	0.000086	0.0084	0.000102
C spine	0.0073	0.000032	0.0118	0.000028
L spine	0.0169	0.000082	0.0216	0.000001
Neck	0.0096	0.000075	0.0133	0.000052
Chest	0.0243	0.000164	0.0271	0.000143
Abdomen & pelvis	0.0155	0.000279	0.0201	0.000275
Coronary angiography	0.0267	0.000416	0.0325	0.000369
Calcium scoring	0.0268	0.000365	0.0327	0.000287
Aortography	0.0209	0.000279	0.0252	0.000283
Pediatric brain (<2 years)	0.0062	0.000199	0.0063	0.000229
Pediatric brain (4–6 years)	0.0046	0.000271	0.0045	0.000254
Pediatric brain (9–11 years)	0.0031	0.000151	0.0030	0.000199
Pediatric brain (13–15 years)	0.0024	0.000194	0.0023	0.000185
